# How the diversity in digestion in carnivorous plants may have evolved

**DOI:** 10.1111/nph.70229

**Published:** 2025-05-28

**Authors:** Andrej Pavlovič

**Affiliations:** ^1^ Department of Biophysics, Faculty of Science Palacký University Šlechtitelů 27 CZ‐783 71 Olomouc Czech Republic

**Keywords:** carnivorous plants, digestive enzymes, jasmonic acid, pitcher plant, Venus flytrap

## Abstract

Carnivorous plants secrete digestive enzymes for prey degradation. Although carnivorous plants have a polyphyletic origin and evolved several times independently, they surprisingly co‐opted similar digestive enzymes during convergent evolution. However, despite having similar digestive enzymes, the mode of their regulation strongly differs across different phylogenetic lineages. But what factors are responsible for such diversity in their digestion? By combining phylogenetic relationships of digestive fluid proteins and biochemical data, the analyses showed that phylogeny seems to be a significant factor determining the regulation of digestion, but environment (water vs terrestrial) and type of trap do not affect regulation. The oldest carnivorous plant lineage, Caryophyllales, co‐opted phytohormone jasmonic acid (JA) for regulation of digestive enzyme activity. However, the remaining orders of carnivorous plants do not accumulate JA in response to prey capture, and their digestive enzyme activity is not responsive to exogenous JA application. Instead, they use different modes of regulation, for example, development/senescence, osmotically induced and constitutive. These different modes of regulation can be explained by co‐option, albeit of similar genes but different paralogs with different *cis* regulatory elements that have been fine‐tuned during evolution.

## Introduction

Carnivorous plants have the ability to capture, kill, and digest animal prey in specialized traps. They absorb nutrients from the victims and utilize them for plant growth and development (Adamec *et al*., 2021). Carnivorous plants' ability to digest animal prey is most fascinating but has also been shrouded in mystery. It is remarkable that at the beginning of the 21^st^ century, 30 years after the first man landed on the moon's surface, we had no convincing evidence about the origin of digestive enzymes in carnivorous plants: whether they use endogenous digestive enzymes or rely only on symbiotic microorganisms in digestion. Although there were numerous attempts to chromatographically and electrophoretically purify the digestive enzymes mainly from *Nepenthes* pitcher plants (Steckelberg *et al*., 1967; Jentsch, 1972; Tökes *et al*., 1974; Heslop‐Harrison, 1975), doubts about the origin of the enzymes in digestive fluids persisted in the last century (for review, see Frazier, 2000). But the situation changed in 2004, when a group of Japanese scientists, led by Professor Takahashi, isolated and purified to homogeneity the first endogenous digestive enzyme aspartic protease called nepenthesin from *Nepenthes* (Athauda *et al*., 2004). They cloned cDNAs for the enzyme from the pitcher tissue of *Nepenthes gracilis* to deduce the complete amino acid sequence and primary structure determination to confirm that it is indeed a plant‐derived enzyme. Later, a boom in LC‐MS analyses led to the identification of other digestive enzymes in the digestive fluid of carnivorous plants. The analyses found that they are usually related to pathogenesis‐related (PR) proteins, that is, proteins which plants use for defence (Hatano & Hamada, 2008, 2012; Mithöfer, 2011; Lee *et al*., 2016). This was the first convincing evidence for the old hypothesis that botanical carnivory has evolved from a plant defence mechanism, as had been suggested before (Juniper *et al*., 1989). However, after the discoveries of endogenous digestive enzymes, a new question arose: how is the activity and gene expression of the enzymes regulated? And here again the plant defence mechanisms came to the aid of carnivorous plants. Phytohormones from the group of jasmonates (JAs) were discovered to play a significant role in the regulation of digestive enzyme expression in the genera *Dionaea*, *Drosera*, and *Nepenthes* (Libiaková *et al*., 2014; Bemm *et al*., 2016; Yilamujiang *et al*., 2016; Krausko *et al*., 2017; Pavlovič *et al*., 2017; Pavlovič & Mithöfer, 2019). JAs are important regulators in plant responses to biotic and abiotic stresses as well as in development processes (Wasternack & Hause, 2013). Mechanical wounding or herbivore attack is one of the most prominent examples where JAs are involved as a signal (Koo & Howe, 2009). The endogenous rise of JAs upon wounding or herbivore and pathogen attack is associated with the induction of synthesis of secondary metabolites and activation of the defence response (Wasternack & Hause, 2013). Particularly, the bioactive isoleucine conjugate of JA (JA‐Ile) triggers an interaction between the CORONATINE INSENSITIVE1 (COI1) receptor and members of the JASMONATE ZIM‐DOMAIN (JAZ) family of repressors. COI1‐mediated degradation of JAZ repressors activates the reprogramming of gene expression leading to the plant defence response (Chini *et al*., 2007; Thines *et al*., 2007; Fonseca *et al*., 2009; Sheard *et al*., 2010). In carnivorous plants, JA‐Ile activates the digestive process and thus, carnivorous plants co‐opted JA‐mediated plant defence mechanisms for botanical carnivory. However, despite the fact that JAs are ubiquitous for plant defence mechanisms (Chini *et al*., 2023), are they ubiquitous also for botanical carnivory? Recent studies have shown that different carnivorous plant genera employed different modes of regulation of digestion (Nishimura *et al*., 2013; Pavlovič *et al*., 2024; Procko & Chory, 2024). Here, I will discuss how this mode is dependent on phylogeny rather than on trap types and habitats, and I will outline the molecular mechanisms by which different modes of regulation may have evolved.

### Mechanism of prey capture

**Fig. 1:**
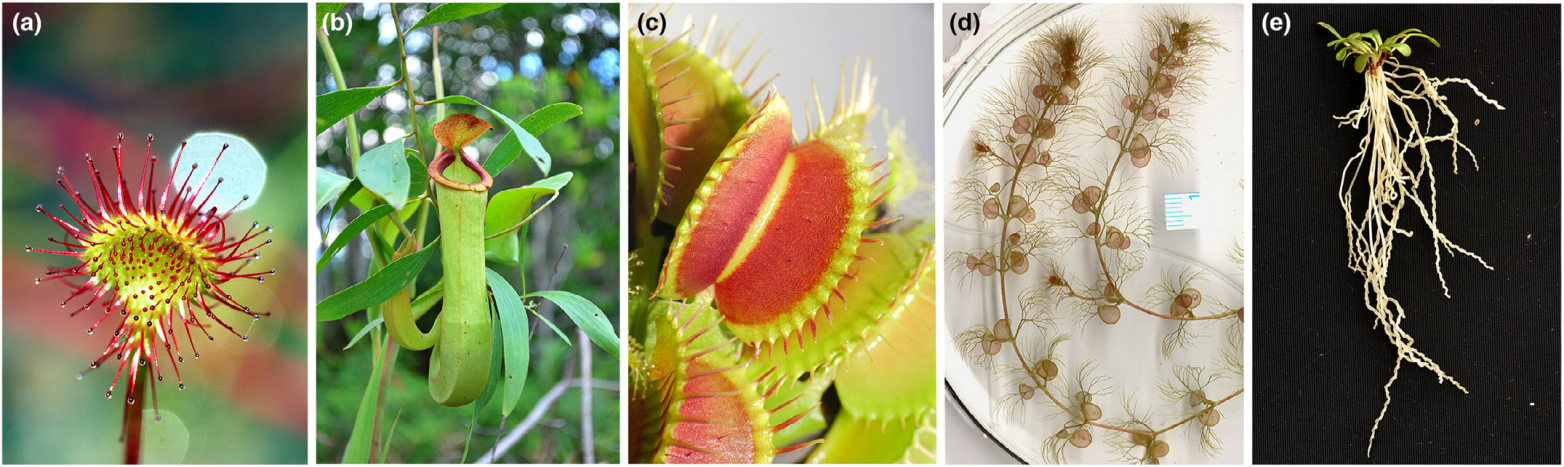
Different types of traps in carnivorous plants. (a) Adhesive trap of sundew *Drosera rotundifolia*, (b) pitcher trap of *Nepenthes mirabilis*, (c) snap trap of Venus flytrap (*Dionaea muscipula*), (d) bladder suction trap of aquatic bladderwort *Utricularia reflexa*, (e) eel trap of *Genlisea hispidula*.

**Fig. 2:**
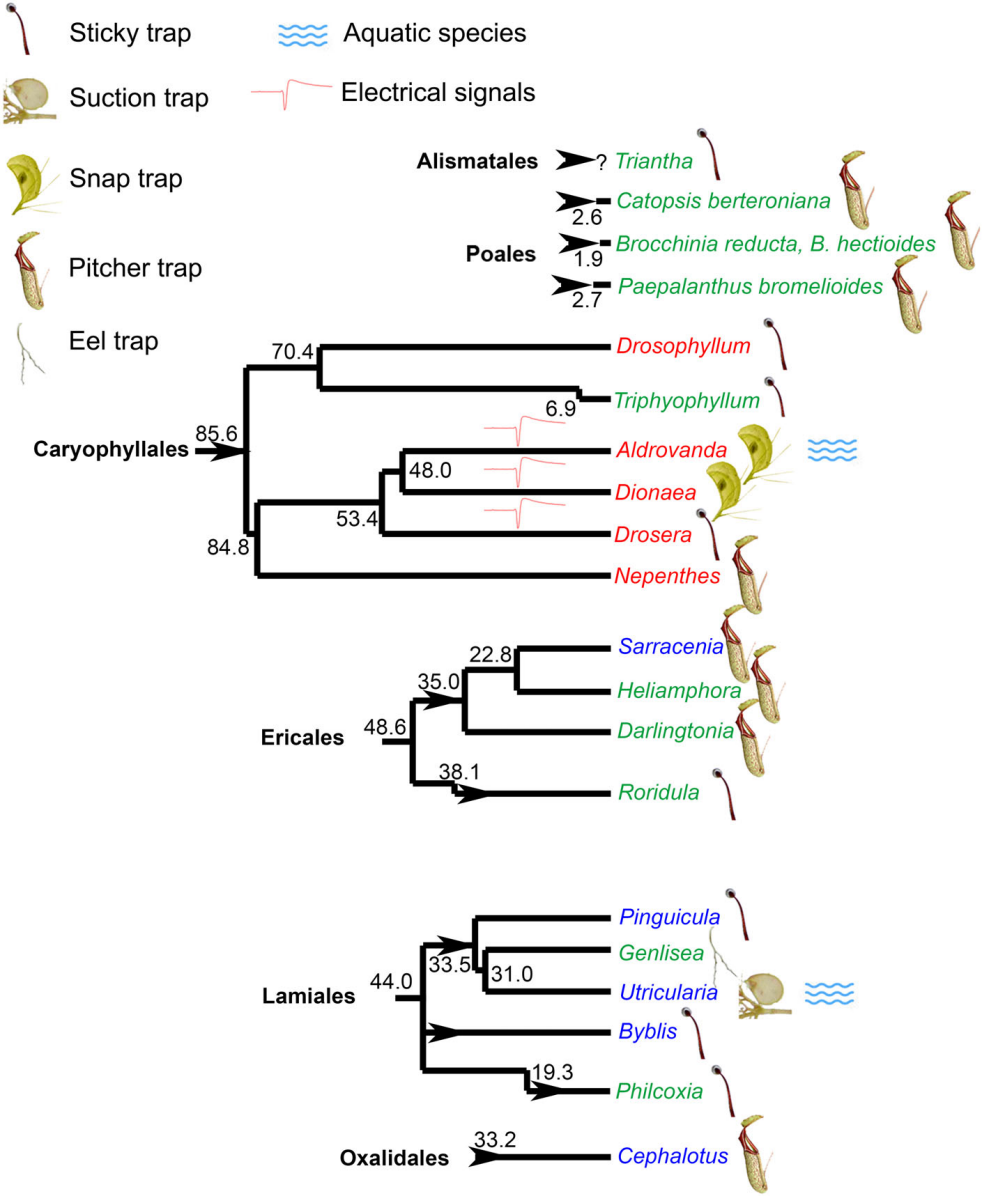
Phylogeny of jasmonate (JAs) signalling in different carnivorous plant lineages. Carnivorous plants have evolved at least 11 times independently (arrowheads) in six orders of angiosperms. The genera of carnivorous plants in which the JAs are involved in the regulation of enzyme secretion are depicted in red. The genera in which JAs do not play any role in the regulation of enzyme activities are depicted in blue. The genera that have not yet been investigated in this respect are depicted in green. The genera that use fast electrical signalling are marked by a symbol of action potential. The genera using JA signalling form a clear cluster in the Caryophyllales order without any association with the type of environment (aquatic/terrestrial) or trap type. The numbers depict estimated phylogenetic age in million years ago according to Fleischmann *et al*. (2018). Adapted from Pavlovič *et al*. (2024).

Carnivorous plants employ five different trapping mechanisms for prey capture: adhesive (‘flypaper’) traps with a sticky glandular surface (e.g. *Drosera*, *Drosophyllum*, *Pinguicula*, and *Byblis*); pitfall (‘pitcher’) traps forming a central cavity or small tanks filled with digestive fluid (e.g. *Nepenthes*, *Sarracenia*, and *Cephalotus*); mobile snap‐traps with rapidly closing lobes (e.g. *Aldrovanda* and *Dionaea*); suction (‘bladder’) traps suck the prey by actively forming negative pressure inside (e.g. *Utricularia*); and specialized eel (‘lobster‐pot’ and ‘cork‐screw’) traps using inward‐pointing hairs to force prey to move towards a digestive organ (e.g. *Genlisea*; Fig. 1; Pavlovič & Saganová, 2015; Adamec *et al*., 2021). These different trap types have evolved as a result of convergent as well as divergent evolution (Thorogood *et al*., 2018). For example, pitfall traps have evolved several times independently in different evolutionary lineages as a result of convergent evolution (*Cephalotus* in Oxalidales; *Sarracenia*, *Darlingtonia*, and *Heliamphora* in Ericales; *Nepenthes* in Caryophyllales; *Brocchinia* and *Catopsis* in Poales). Sticky traps have evolved also several times independently (*Drosera*, *Drosophyllum*, and *Triphyophyllum* in Caryophyllales; *Byblis*, *Pinguicula*, and *Philcoxia* in Lamiales; *Roridula* in Ericales). On the other hand, different trap types have evolved within one evolutionary lineage as a result of divergent evolution (e.g. Caryophyllales; sticky trap in *Drosera*, *Drosophyllum*, and *Triphyophyllum*; pitfall traps in *Nepenthes*; snap traps in *Aldrovanda* and *Dionaea*). The results we currently have indicate that JA signalling is suitable for botanical carnivory in different trap types: in sticky traps of *Drosera* and *Drosophyllum* (Krausko *et al*., 2017; Pavlovič *et al*., 2024); in snap traps of *Dionaea* and *Aldrovanda* (Libiaková *et al*., 2014; Jakšová *et al*., 2021); and in pitfall traps of *Nepenthes* (Fig. 2, Buch *et al*., 2015; Yilamujiang *et al*., 2016; Saul *et al*., 2023). Although all these traps employ JAs for regulation of digestive enzyme activity, the degree of JA dependence is determined by trap types. For example, the snap traps of *Dionaea* and *Aldrovanda* are completely dependent on JAs and they do not produce any digestive enzymes and fluid without prey stimuli (Nishimura *et al*., 2013; Libiaková *et al*., 2014; Jakšová *et al*., 2021). Sticky traps of *Drosera* and *Drosophyllum* contain a small amount of digestive enzymes already in noninduced sticky fluid without any captured prey (Krausko *et al*., 2017; Pavlovič *et al*., 2024) and at least in *Drosera*, their expression is tissue specific with predominant expression in tentacles in comparison with other plant organs (Nishimura *et al*., 2013; Arai *et al*., 2021). This tissue specificity is regulated by promoter methylation status; that is, in carnivorous organs and glands the promoter is unmethylated suggesting that the promoters do not form a closed chromatin structure and in the presence of transcription activators (or rather absence of JAZ repressor based on our knowledge about JA‐signalling, Thines *et al*., 2007) can be transcriptionally active (Nishimura *et al*., 2013; Arai *et al*., 2021). This basal promoter activity and transcription of digestive enzyme genes can be further strongly enhanced by prey‐induced JA accumulation (Krausko *et al*., 2017; Pavlovič *et al*., 2024). The pitcher plants *Nepenthes*, which probably evolved from sticky traps within Caryophyllales (Thorogood *et al*., 2018), are probably the least dependent on JAs. The pitcher fluid contains many digestive enzymes even in closed immature pitchers and protein levels increase dramatically during pitcher maturation without any prey stimuli (Eilenberg *et al*., 2006; Hatano & Hamada, 2008). The level of digestive enzymes in the fluid is autoregulated as indicated by protein and fluid depletion experiments (Miguel *et al*., 2019; Wan Zakaria *et al*., 2019; Goh *et al*., 2020). Nevertheless, prey stimuli can enhance the expression and secretion of many digestive enzymes through JAs in *Nepenthes* (Buch *et al*., 2015; Yilamujiang *et al*., 2016; Saul *et al*., 2023). This may indicate that the expression of digestive enzymes in *Nepenthes* is under the control of more regulatory elements and its JA and prey‐induced regulation is more relaxed in comparison with *Dionaea*. On the other hand, the same trap types in different genera of carnivorous plants regulate digestive enzyme activity independently of JAs (e.g. sticky traps of *Byblis* and *Pinguicula*, pitfall traps of *Cephalotus* and *Sarracenia*). Some of them show continuous enzyme secretion and the enzymes accumulate over time during pitcher ontogeny without any prey stimuli (e.g. *Sarracenia*), whereas others activate enzyme secretion in response to prey capture by an unknown mechanism (e.g. *Byblis*, *Pinguicula*; Kocáb *et al*., 2020; Pavlovič *et al*., 2024, 2025). Although *Pinguicula* can accumulate JAs in response to prey capture, probably as a result of general trichome physiology (Kocáb *et al*., 2020; Matsumura *et al*., 2022; Sun *et al*., 2024), its digestive enzymes are probably not JA responsive, as evidenced by experiments with exogenous coronatine (i.e. agonist of JA‐Ile signal) application (Kocáb *et al*., 2020).

Thus, in summary, the mechanism of prey capture does not determine the involvement of JAs in the regulation of enzyme secretion but may slightly fine‐tune it (Fig. 2). Where the prey capture is a dynamic process, like rapid trap movement in *Dionaea* and *Aldrovanda*, there was probably a strong positive selection pressure for co‐option and conserving the JA signalling pathway for rapid regulation of the digestive process, which is also involved in rapid JA‐mediated plant defence. Where there is a passive mechanism for prey capture, where the prey slowly accumulate and decompose over time in pitcher traps, there has probably been a relaxation of functional constraints on JAs (e.g. *Nepenthes* which has probably evolved from sticky traps; Thorogood *et al*., 2018) or JA has not been co‐opted at all (e.g. *Sarracenia*; Pavlovič *et al*., 2024), and digestive enzymes might additionally be regulated also JA‐independently driven by osmotic changes in traps, pitcher ontogeny, autoregulatory mechanisms, or by unknown mechanisms.

### Aquatic vs terrestrial habitats

Approximately 60 out of 810 species of carnivorous plants are submerged aquatic plants, comprising the monotypic *Aldrovanda vesiculosa* and dozens of *Utricularia* species (Adamec *et al*., 2021). Although the remaining carnivorous plants are terrestrial, they often grow in wet environments, often rooted in waterlogged soil, and can even be temporarily seasonally submerged. Some carnivorous plants can tolerate seasonally dry environments (e.g. *Drosophyllum lusitanicum*) but they often give up carnivory temporarily (e.g. Mexican *Pinguicula*, Australian pygmy *Drosera*; Paniw *et al*., 2017; Pavlovič, 2022). A recent study has shown that the submergence of *Arabidopsis thaliana* plants deactivates wound‐induced plant defence against herbivores mediated by JAs (Lee *et al*., 2020). This finding motivated us to perform experiments with aquatic carnivorous plants, and we found that aquatic *A. vesiculosa*, which is sister to terrestrial *D. muscipula*, is fully able to activate the JA signalling pathway for the regulation of digestive enzyme activity. It clearly accumulated JAs in response to feeding, and digestive enzymes responded to their exogenous application (Jakšová *et al*., 2021). It has also strongly expanded genes encoding JA signalling in its genome (Palfalvi *et al*., 2020). By contrast, aquatic *Utricularia reflexa* does not use JAs for the regulation of enzyme activity (Jakšová *et al*., 2021), and the expression of digestive enzymes in *Utricularia* is probably constitutive (Sirová *et al*., 2003) and tissue specific, with high expression in traps in comparison with shoots and inflorescence (Lan *et al*., 2017). Following the Lee *et al*. (2020) protocol, we artificially flooded the terrestrial sister of *Aldrovanda*, *Dionaea* plants, and investigated their ability to trigger the digestive process in an aquatic environment. *Dionaea* plants can often grow in submerged environments in natural habitats. Repeated mechanical stimulation under the water triggered hermetical trap sealing, accumulation of JA and JA‐Ile, and expression of digestive enzymes, but to a lesser extent (Jílková, 2023). This suggests, combined with the fact that its sister genera *Aldrovanda* retained its use of JA signalling for carnivory, that an aquatic environment is not an obstacle for using JA signalling. *Aldrovanda vesiculosa* probably entered the aquatic environment already as a carnivorous plant (Cameron *et al*., 2002), and JA signalling was retained during this transition. Even on the opposite moisture scale, the most drought‐tolerant carnivorous plant, *Drosophyllum lusitanicum* (Paniw *et al*., 2017), co‐opted JAs as its aquatic relative *Aldrovanda* (Pavlovič *et al*., 2024). Thus, the water environment was probably not an important selection factor determining JA signalling for the regulation of botanical carnivory (Fig. 2).

### Phylogeny

Carnivorous plants have a polyphyletic origin and have evolved at least 11 times independently in a time span of 95.1–1.9 Mya (Fleischmann *et al*., 2018) in 6 orders, 13 families, and 20 genera (Adamec *et al*., 2021 and updated by the new genera *Triantha*, Lin *et al*., 2021). Studies on the regulation of digestive enzyme activity in carnivorous plants during the last decade indicate that JAs have been co‐opted probably only once in the oldest order of carnivorous plants, Caryophyllales (Fig. 2, Pavlovič *et al*., 2024). These plants include terrestrial (e.g. *Dionaea*) as well as aquatic carnivorous plants (e.g. *Aldrovanda*) with different trapping mechanisms (e.g. sticky traps in *Drosera* and *Drosophyllum*; snap traps in *Dionaea*; and pitcher traps in *Nepenthes*) as mentioned above. Although similar trapping strategies and the same digestive enzymes also occur in the remaining evolutionary lineages of carnivorous plants (Fukushima *et al*., 2017), it seems that JAs have never been recruited again. The pitcher traps of *Sarracenia* (order: Ericales) and *Cephalotus* (order: Oxalidales) rely more on developmental combined with water‐induced regulation (i.e. osmotic regulation) and constitutive enzyme secretion, respectively (Gallie & Chang, 1997; Nishimura *et al*., 2013; Pavlovič *et al*., 2024). So, how is it possible that the same digestive enzymes are regulated so differentially in different evolutionary lineages of carnivorous plants?

**Fig. 3:**
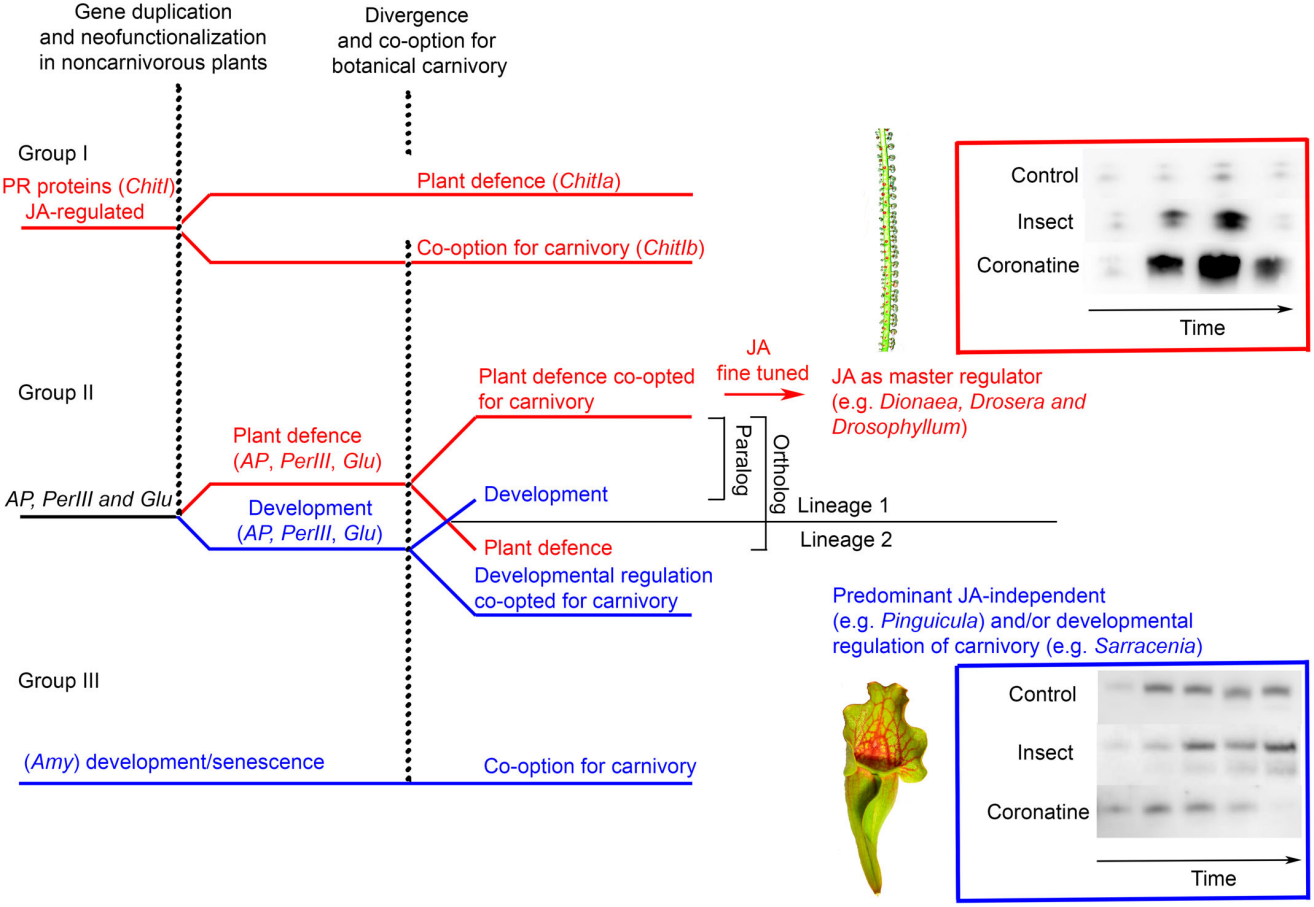
Hypothesis of how different modes of regulation of digestive enzyme activity in carnivorous plants may have evolved. Some genes (group I) encode pathogenesis‐related (PR) proteins, which were already jasmonate responsive in non‐carnivorous plants, for example type I chitinase (*ChitI*). They could undergo duplication and subfunctionalization for pathogenic response (subclass Ia) and carnivory (subclass Ib) as is evident in the Caryophyllales order (Renner & Specht, 2012, 2013). On the other hand, type III chitinase was probably retained as a single gene for both functions (not shown as we still have few data about its JA regulation, Rotloff *et al*., 2011). Other genes (group II) encode enzymes with a broad spectrum of biological roles which evolved by gene duplications and their functional divergence. One copy of the gene retained its function and a new copy of the gene acquired a new function. Different lineages of carnivorous plants co‐opted different paralogs of genes encoding, for example, aspartic protease (*AP*), class III peroxidase (*PerIII*) and β‐1,3‐glucanases (*Glu*) for carnivory with different sets of *cis*‐regulatory elements; some of them were involved in defence and some of them in development including senescence and/or developmentally or constitutively regulated defence. They could be retained as a single copy gene for both functions or they (neo‐)subfunctionalized. They were probably also adjusted and fine‐tuned lineage specifically later in evolution, which may explain that in genera of the Caryophyllales order, the digestive enzymes are under the control of JA and in the orders Ericales, Lamiales and Oxalidales are regulated JA‐independently (e.g. developmentally, constitutively). Other genes (group III) encode enzymes involved in plant development like α‐amylase (*amy*) and are examples of genes with JA‐independent regulation. So far we have still very little information about their molecular evolution in carnivorous plants. Plant JA‐regulated defence‐related genes are shown in red, and developmentally regulated genes are shown in blue. The insets show Western blots of secreted aspartic protease into the digestive fluid of *Drosophyllum lusitanicum*, which is JA‐responsive (top) and *Sarracenia purpurea*, which is not (bottom), regulating enzyme secretion rather developmentally (according to Pavlovič *et al*., 2024). The simplified scheme. [Correction added on 7 June 2025, after first online publication: the word ‘Speciation’ was updated to ‘Divergence’ within Fig. 3].

Now based on the studies on the Caryophyllales order, there is a consensus that carnivorous plants co‐opted plant defence mechanisms for botanical carnivory because many digestive enzymes found in the secretome of carnivorous plants are involved in plant defence reactions in noncarnivorous plants (e.g. aspartic and cysteine proteases, chitinases, class III peroxidases, and β‐1,3‐glucanases; Mithöfer, 2011; Bemm *et al*., 2016). However, this view on botanical carnivory can be oversimplified. Whereas some genes are indeed involved in plant defence mechanisms and belong to the PR‐proteins (e.g. chitinases, Loon *et al*., 2006) and are strongly induced by pathogen/herbivore attack and defence‐related hormones (i.e. JA, Dávila‐Lara *et al*., 2021), others, for example aspartic proteases, are a group of very diverse proteolytic enzymes with a broad spectrum of biological roles, with, for example, 78 genes encoding putative aspartic proteases in Arabidopsis (Yu & Feng, 2025). Plant aspartic proteases are suggested to undergo functional specialization and to be crucial in developmental processes, as well as in biotic and abiotic stress responses (Simões & Faro, 2004; Figueiredo *et al*., 2021; Yu & Feng, 2025). In carnivorous plants, aspartic proteases have been co‐opted for botanical carnivory several times independently from paralogous gene lineages, mainly from atypical, nucellin‐like aspartic proteases (Athauda *et al*., 2004; Fukushima *et al*., 2017; Soares *et al*., 2019) and also from saposin‐like aspartyl protease (e.g. *Cephalotus*; Fukushima *et al*., 2017). Because *cis*‐regulatory elements are usually conserved in orthologous gene promoter sequences across diverging species, it means that whereas some lineages might indeed have co‐opted homologs of aspartic proteases that are induced by pathogen/herbivore attack and were further improved for more stringent JA control (e.g. Caryophyllales), others might have co‐opted homologs of aspartic proteases involved in development/senescence (or developmentally‐regulated defence) and thus with different regulation (Fig. 3). For example, the closest orthologs to the aspartic protease found in the digestive fluid of *S. purpurea* in *A. thaliana* are aspartic protease CHLOROPLAST NUCLEOID DNA BINDING PROTEIN 41 (CND41, AT5G10770) and APOPLASTIC, EDS1‐DEPENDENT 1 (AED1, AT5G10760), which clearly show developmental, osmotic regulation and a role in senescence (Kato *et al*., 2004; Fukushima *et al*., 2017). Similarly, the closest ortholog to the aspartic protease found in the digestive fluid of *C. follicularis* in *A. thaliana* is CONSTITUTIVE DISEASE RESISTANCE (CDR1, AT5G33340, Fukushima *et al*., 2017) which shows rather constitutive expression and salicylic acid (SA)‐induced response (Xia *et al*., 2004). These orthologs also do not show significant insect‐stimulated expression in *Dionaea* (see in Rentsch *et al*., 2024, and for expression profile of Arabidopsis orthologs see The Arabidopsis Information Resource (TAIR) or Fukushima *et al*. (2017)). On the other hand, the closest relative ortholog of the aspartic protease to *Dionaea* and *Nepenthes* in *A. thaliana* (AT2G03200) exhibits mild JA regulation (Fukushima *et al*., 2017), which was probably further improved during evolution. This may explain why the expression and secretion of aspartic proteases are regulated developmentally and osmotically in *S. purpurea*, are rather constitutive in *C. follicularis*, and are regulated by JA in the Caryophyllales order (Pavlovič *et al*., 2017, 2024). The same situation is also clear for β‐1,3‐glucanases. Although research on β‐1,3‐glucanases to date has focused primarily on their PR function, β‐1,3‐glucanases also play critical roles in normal developmental plant processes (Doxey *et al*., 2007). The closest relative orthologs of β‐1,3‐glucanase to four species of carnivorous plants in *A. thaliana* exhibit functions not only in plant defence (At3g57260) but also in pollen maturation (At3g57270) (Doxey *et al*., 2007; Michalko *et al*., 2017). The next example is class III peroxidases which are also involved not only in the defence response but also in lignification, cell elongation, and seed germination (Shigeto & Tsutsumi, 2016). The closest relative orthologs of class III peroxidase to the carnivorous plant *Cephalotus* in *A. thaliana* (At5g58400, At5g58390) do not exhibit strong induction in response to pathogen attack in contrast to other paralogs (e.g. At5g05340; Fukushima *et al*., 2017). The closest relative ortholog of type I chitinase to carnivorous *Drosera* and *Dionaea* in *A. thaliana* (AT3g12500) is involved in the ethylene/JA‐mediated signalling pathway during systemic acquired resistance, in contrast to *Cephalotus* (AT3g54420). From these examples, it is obvious that some of the genes encoding digestive enzymes in carnivorous plants are regulated similarly to their orthologs in noncarnivorous plants. But there are also indications that the regulation of some of the genes was adjusted later in evolution. Because the promoters of genes encoding digestive enzymes contain several regulatory *cis*‐acting elements (Arai *et al*., 2021; Libantová *et al*., 2021; Mikitová *et al*., 2025), it is highly probable that they were adjusted lineage specifically. Nutrient transporters are usually constitutively expressed in roots; however, in carnivorous Droseraceae, they are induced by prey and JAs (Palfalvi *et al*., 2020). Indeed, co‐option may involve the addition of novel *cis*‐regulatory elements (True & Carroll, 2002). Palfalvi *et al*. (2020) found that genes associated with carnivory in Droseraceae are under the control of WRKY transcription factors, which exhibit sub‐functionalization following the duplication event. WRKY transcription factors often act as a positive regulator in JA‐mediated plant defence in noncarnivorous plants and could play an important role in the recruitment of genes to carnivory‐related functions in Caryophyllales (Palfalvi *et al*., 2020). Indeed, the promoter region of genes encoding digestive enzymes in Droseraceae is enriched in WBOX sequences (Arai *et al*., 2021, Libantová *et al*., 2021, Mikitová *et al*., 2025). For example, the number of WBOX sequences (WRKY binding site) in the promoter region of S‐like RNAse in *Dionaea* is four times higher than in *Cephalotus* (Nishimura *et al*., 2013).

In contrast to the above‐mentioned enzymes, the enzymes involved in phosphate utilization, RNAses and acid phosphatases, showed unexpected orthologous relationships among nonrelated carnivorous plants with convergent amino acid changes (Nishimura *et al*., 2014; Fukushima *et al*., 2017). So, even when different evolutionary lineages co‐opted different paralogs, they were under the strong selective pressure of a carnivorous lifestyle and accumulated convergent amino acid changes (Nishimura *et al*., 2014; Fukushima *et al*., 2017). But despite convergent amino acid changes in the coding region, they are also regulated differentially (Nishimura *et al*., 2013), indicating that promoters were under different selection pressure in comparison with the coding region of the gene in different lineages and/or trap types.

Recently, we identified the unique enzyme α‐amylase found in the digestive fluid of butterwort *Pinguicula*, which was not induced by JAs (Kocáb *et al*., 2020). The α‐amylases are enzymes that are mainly involved in initiating starch degradation and plant development (Stanley *et al*., 2005). There are three families of α‐amylases in plants differing in their cellular localization. None of these proteins are more than 48% identical to each other and are thus not likely to have arisen from recent gene duplications. Family 1 is secretory; family 2 has no predicted targeting peptide, and therefore is thought to localize to the cytoplasm; and family 3 contains a predicted chloroplast transit peptide. Family 1 contains well‐characterized cereal grain α‐amylases secreted from the cells of the aleurone layers to the endosperm promoting germination (Stanley *et al*., 2005). The presence of this enzyme in the secretome of carnivorous plants has not been found in any other genera of carnivorous plants before and is unique for *Pinguicula*. The function of family 1 in dicotyledons is much less understood than in cereals, but in the leaf of *A. thaliana*, the AT4g25000 ortholog is induced by different biotic and abiotic stresses (induced by abscisic acid, ABA; but not JAs) and senescence (Stanley *et al*., 2005; Doyle *et al*., 2007). The fact that the Arabidopsis ortholog AT4g25000 is strongly induced by osmotic stress is in line with evidence of osmotically driven digestive fluid secretion mediated by Cl^−^ influx to the head cells of digestive glands in *Pinguicula*, induced by prey (Heslop‐Harrison & Heslop‐Harrison, 1980). The same is also known in *D. muscipula* (Rea *et al*., 1983) and the hyperosmotic salinity response was among the enriched gene ontology (GO) terms in insect and coronatine stimulated traps in RNA‐seq experiments (Bemm *et al*., 2016). The possible regulatory effect of osmotic changes in the glands of carnivorous plants needs further experimentation.

Thus, co‐option of different paralogs of hydrolytic enzymes regulated by different hormones (e.g. ABA, SA, and indole‐3‐acetic acid (IAA)), development, osmotic changes, or senescence can explain their different regulation in different lineages of carnivorous plants and are motivating for the search of other regulatory elements involved in botanical carnivory. This however does not rule out the possibility that many of these paralogs are also involved in plant defence, because, for example, plant senescence is the developmental process leading to nutrient remobilization and finally the degradation and death of tissue and can be triggered by many pathogens (Häffner *et al*., 2015). Thus, co‐option of defence‐related genes has been probably the main driving force for evolution of botanical carnivory. But why only the oldest lineage of carnivorous plants co‐opted the plant defence hormone JA as a master factor for regulation of carnivory is unclear, as are the environmental factors which were the driving force for such co‐option in Caryophyllales 90 Mya ago. It is quite interesting that the other well‐known plant defence hormone SA has not been found to regulate digestive enzyme expression in *Drosera*, *Nepenthes*, or *Pinguicula* (Matušíková *et al*., 2005; Buch *et al*., 2015; Krausko *et al*., 2017; Kocáb *et al*., 2020). Moreover, no other phytohormone has been shown to be able to trigger the expression of digestive enzymes except JA in carnivorous plants and this issue is open to further investigation. Also phytohormone crosstalk, which is crucial for adjusting plant responses to environmental stimuli in different situations, is highly understudied in carnivorous plants. The antagonism between ABA and JA in *Dionaea*, and SA and JA in *Drosera* in regulating excitability and enzyme secretion, respectively, has already been described (Escalante‐Pérez *et al*., 2011; Krausko *et al*., 2017), as well as possible synergism between JA and IAA in trap folding reaction in *D. capensis* plants (La Porta *et al*., 2019).

## Future perspectives

**Fig. 4:**
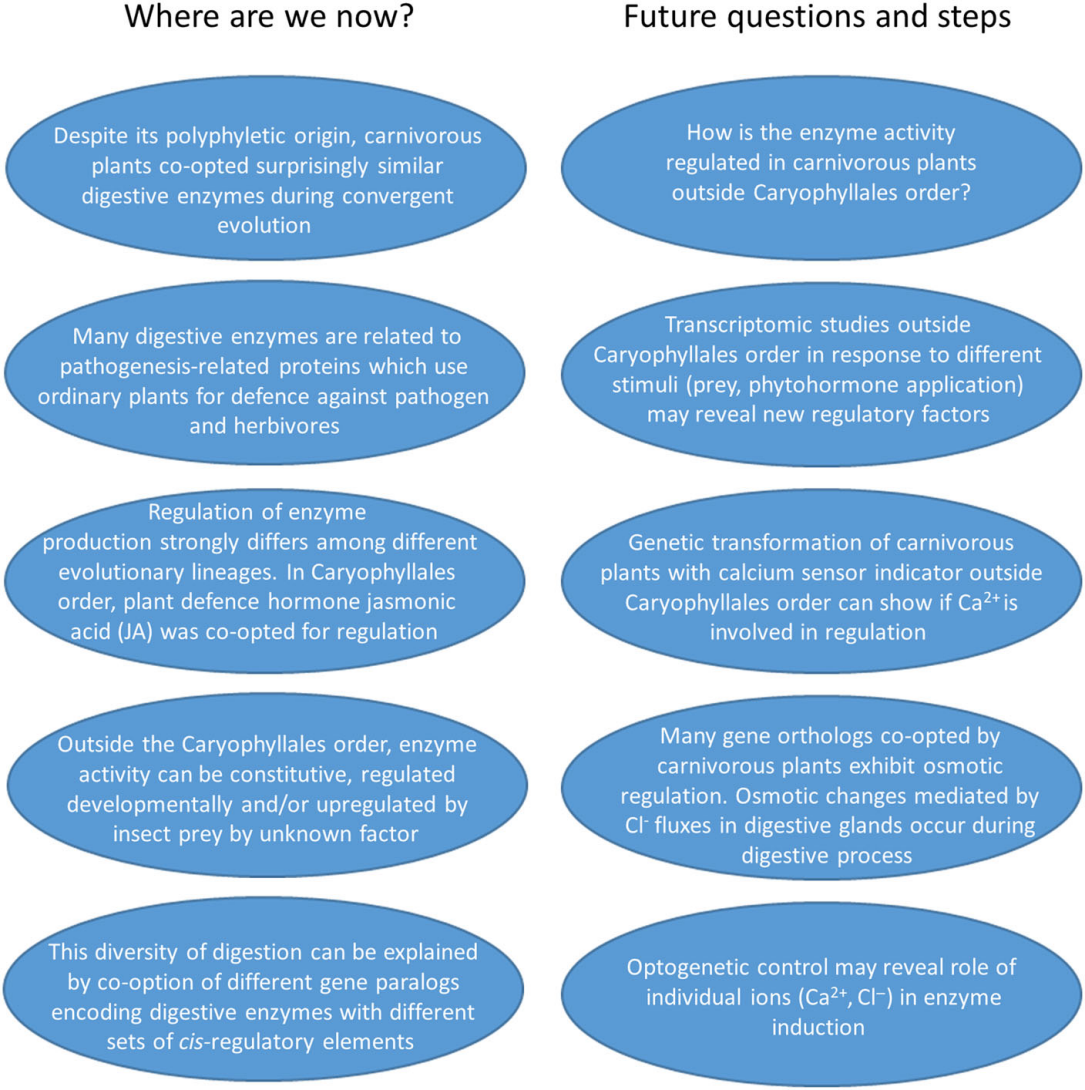
Box showing what we know and future questions and steps in the research on the regulation of enzyme expression and secretion in carnivorous plants.

JAs are important regulators of botanical carnivory in the oldest lineage of carnivorous plants, Caryophyllales, where they were co‐opted from plant defence mechanisms. The speed of response in plant defence mechanisms is mediated by JAs, which can accumulate very quickly in response to herbivore or pathogen attack (Kimberlin *et al*., 2022). This speed is also very important in capturing and retaining insect prey in carnivorous plants, so it is not surprising that the fastest carnivorous plants *Aldrovanda*, *Dionaea*, and *Drosera* have co‐opted JAs for fast response to prey. But now it has become obvious that co‐option of JA signalling is the exception rather than the rule, and many carnivorous plants regulate enzyme activity differently. Co‐option of different paralogs with different *cis*‐regulatory elements might provide genetic background for further fine‐tuning of regulatory properties. Ontogeny and autoregulating mechanisms are important in the regulation of enzyme expression in some groups of carnivorous plants with passive traps, but which signalling molecules are involved in other genera, which strongly induce enzyme activity in response to prey capture, remains unknown. These plants usually possess glandular trichomes (e.g. *Byblis* and *Pinguicula*), which in noncarnivorous plants act as mechanosensors through Ca^2+^ signalling (Matsumura *et al*., 2022; Sun *et al*., 2024). Although Ca^2+^ signal is also important for the initiation of JA response in carnivorous plants within the Caryophyllales order (Suda *et al*., 2020; Procko *et al*., 2022), there are many examples where Ca^2+^ acts independently of JAs (e.g. calmodulin‐binding transcription activator (CAMTA)‐dependent pathway; Darwish *et al*., 2022; Matsumura *et al*., 2022). Whether this general trichome physiology has been co‐opted for botanical carnivory outside the Caryophyllales order is not clear. Also, chemical signals (e.g. protein and chitin) from insect prey may play an important role, as is known in the Caryophyllales order. How these signals are recognized by carnivorous plants is not clear (Bemm *et al*., 2016; Jakšová *et al*., 2020). There is also a possibility that the secretion of digestive enzymes can be driven by osmotic changes in digestive glands, as the expression of many *Arabidopsis* orthologs is regulated by osmotic changes, and rapid Cl^−^ uptake in digestive glands, followed by the flow of water, occurs in response to prey capture (Heslop‐Harrison & Heslop‐Harrison, 1980; Rea *et al*., 1983). Such osmotic regulation has not been investigated in carnivorous plants so far but has been shown to play a role in the release of presynthesized enzymes from gland cell walls (Heslop‐Harrison & Knox, 1971; Kocáb *et al*., 2020). Recent success in the dissection of Ca^2+^ and Cl^−^ signalling optogenetically (Ding *et al*., 2024; Hedrich & Gilliham, 2025) may be useful for the investigation of related processes in carnivorous plants, as both ions play a role in the initiation of digestion (Heslop‐Harrison & Heslop‐Harrison, 1980; Rea *et al*., 1983; Suda *et al*., 2020). Transformation of carnivorous plants from different orders outside the Caryophyllales order by calcium sensor indicators and channelrhodopsins for optogenetic control can help answer these questions. The problems in the past were the recalcitrance of carnivorous plants and the difficulty in their transformation and generation of transgenic and mutant plants, which have recently been overcome (Oropeza‐Aburto *et al*., 2020; Suda *et al*., 2020; Procko *et al*., 2022, 2023; Coronado‐Martín *et al*., 2024). Also, the database of digestive enzyme sequences is growing (Fukushima *et al*., 2017). These genetic tools may become very useful in further studies focused on digestion regulation in carnivorous plants. The classic approach using phytohormonics, proteomics, and transcriptomics has become very useful to unravel the involvement of JAs in the regulation of botanical carnivory and can also be helpful in future studies. Mainly, transcriptomic studies outside the Caryophyllales in response to the application of different stimuli (e.g. prey and phytohormones) can be important when looking for a new regulatory mechanism (Fig. 4).

## Competing interests

None declared.

## Disclaimer

The New Phytologist Foundation remains neutral with regard to jurisdictional claims in maps and in any institutional affiliations.
